# Use of Starter Cultures in Foods from Animal Origin to Improve Their Safety

**DOI:** 10.3390/ijerph18052544

**Published:** 2021-03-04

**Authors:** Juan García-Díez, Cristina Saraiva

**Affiliations:** 1 CECAV—Animal and Veterinary Research Centre, University of Trás-os-Montes e Alto Douro, Quinta de Prados, 5001-801 Vila Real, Portugal; 2 Department of Veterinary Sciences, School of Agrarian and Veterinary Sciences, University of Trás-os-Montes e Alto Douro, Quinta de Prados, 5001-801 Vila Real, Portugal; crisarai@utad.pt

**Keywords:** starter cultures, foodborne pathogens, fermented meats, cheese, yogurt, fermented fish, microbial food safety, chemical food safety

## Abstract

Starter cultures can be defined as preparations with a large number of cells that include a single type or a mixture of two or more microorganisms that are added to foods in order to take advantage of the compounds or products derived from their metabolism or enzymatic activity. In foods from animal origin, starter cultures are widely used in the dairy industry for cheese, yogurt and other fermented dairy products, in the meat industry, mainly for sausage manufacture, and in the fishery industry for fermented fish products. Usually, microorganisms selected as starter culture are isolated from the native microbiota of traditional products since they are well adapted to the environmental conditions of food processing and are responsible to confer specific appearance, texture, aroma and flavour characteristics. The main function of starter cultures used in food from animal origin, mainly represented by lactic acid bacteria, consists in the rapid production of lactic acid, which causes a reduction in pH, inhibiting the growth of pathogenic and spoilage microorganisms, increasing the shelf-life of fermented foods. Also, production of other metabolites (e.g., lactic acid, acetic acid, propionic acid, benzoic acid, hydrogen peroxide or bacteriocins) improves the safety of foods. Since starter cultures have become the predominant microbiota, it allows food processors to control the fermentation processes, excluding the undesirable flora and decreasing hygienic and manufacturing risks due to deficiencies of microbial origin. Also, stater cultures play an important role in the chemical safety of fermented foods by reduction of biogenic amine and polycyclic aromatic hydrocarbons contents. The present review discusses how starter cultures contribute to improve the microbiological and chemical safety in products of animal origin, namely meat, dairy and fishery products.

## 1. Introduction

Starter cultures can be defined as preparations with a large number of cells, either of a single type or a mixture of two or more microorganisms that are added to foods in order to take advantage of the compounds or products derived from their metabolism or enzymatic activity [1].

Since starter cultures are used to perform fermentation processes in food production, its use is a common practice in the food industry worldwide. This has resulted in the commercialisation of several products such as bioprotective cultures, starters or probiotics aimed to provide foods with specific sensory and nutritional characteristics, potential health benefits and guarantee food safety [2].

Starter cultures are used in a wide range of food industries such as the dairy industry for cheese, yogurt and other fermented dairy products’ manufacture [3], the meat industry, mainly for sausage manufacture [4], alcohol production for the beer and wine industry [5,6], vinegar production [7], preparation of oriental products based on rice and soy [8], baking, fermented cereals [9] and production of fermented fruits and vegetables [10,11,12]. Since starter cultures are adapted to the substrates, they allow us control of the fermentation process to obtain predictable results [13].

The most promising microorganisms selected as starter culture are those that are isolated from the native microbiota of traditional products [14] since they are well adapted to the environmental conditions of food and are capable of controlling spoilage and pathogenic microbiota of food [15].

To select a microorganism(s) as a starter or starter culture, it is necessary to carry out a proper study regarding its metabolism and activities, since in some cases, its effects and/or properties may vary between laboratory conditions and food products [16]. Also, starter culture must be recognised as safe, capable of being produced on a large scale and remain viable and stable during storage [17].

Microorganisms used as starter cultures are bacteria, moulds and yeast. Within the group of bacteria, lactic acid bacteria (LAB) are the most representative group, being used in fermentation processes of meat and dairy products [18]. In addition, other bacterial groups such as Gram-positive, catalase-positive cocci, mainly coagulase-negative staphylococci (CNS), and *Micrococcaceae* are also used [19,20]. Yeasts are mainly used for the fermentation of alcoholic beverages [21], with wine and beer production being the most representative. Regarding starter moulds, they are used to obtain fermented vegetable products, cheeses and meat products [22].

The present work discusses how starter cultures contribute to improve the food safety in products of animal origin, namely meat, dairy and fishery products.

## 2. Use of Starter Cultures to Improve the Food Safety in Fermented Meat Products

Fermented meat products represent the oldest known way of preserving meat to achieve a microbiologically stable product with particular sensory characteristics that can be kept for several months [23]. Fermented meat sausages are products that are made with minced meat and fat mixed with salt, spices and authorised additives which are mixed and stuffed into natural or artificial casings and subjected to a drying process in which a microbial fermentation takes place, resulting in a drop of pH and water activity (aW) levels [24]. Traditionally, the fermentation process of these meat products is developed by the natural microbiota existing in meat. However, the use of commercial starter cultures is currently widespread in the meat industry. Starter cultures can be defined as microorganisms selected according to their specific properties that are added to meat batter to improve some characteristics such as appearance, texture, aroma and flavour. Use of starter cultures enables homogenisation of production and avoids possible defects. In addition, they improve the safety of fermented meat products by production of several compounds such as lactic acid, acetic acid, propionic acid, benzoic acid, hydrogen peroxide or bactericidal proteins (i.e., bacteriocins), among others [25]. Thus, starter cultures become the predominant microbiota, directing the fermentation and excluding the undesirable flora, decreasing hygienic and manufacturing risks due to deficiencies of microbial origin.

Regarding organic acids, they inhibit spoilage and foodborne pathogens mainly by reduction of pH. The acid environment interferes with the maintenance of the cell membrane that alters both the structure and functionality, leading to cell death. The antimicrobial effect of organic acids in food has been investigated [26,27,28]. Thus, acetic acid is used to inhibit the growth of both Gram-positive and Gram-negative bacteria, yeasts and fungi. Its inhibitory effect is more pronounced at low pH and presented special importance in fermented vegetables and vinegar industry but is less interesting in foods of animal origin [29]. Benzoic acid occurs naturally in fermented milk products (e.g., kefir, yogurt), produced by microorganisms such as *Lactobacillus acidophilus*, *Lacticaseibacillus casei* or *Lactobacillus helveticus* [30]. Its antimicrobial effect against *Staphylococcus aureus* and *Pseudomonas* has been recently evaluated [31]. Antimicrobial effect of diacetyl, acetic acid and propionic acid against *Salmonella typhimurium*, *Escherichia coli*, *S. aureus* and *Listeria monocytogenes* has also been evaluated [32,33]. Regarding phenyllactic acid, produced by several LAB genera [34], it displayed both bactericidal (against *L. monocytogenes* and *S. aureus*) [35] and antifungal effects [36]. As described, the organic acids produced by LAB contribute to the safety of foods by creating an adverse environment (by low pH) that interferes in the cell membrane permeability. However, it is important to highlight that research about the antimicrobial effect of these organic acids has been carried out by addition as a “natural additive” and not during fermentation processes in foods. Indeed, based on the low quantity of organic acid produced [30], the inhibitory effect of these organic acids may result from the synergistic action together with other metabolites produced by starters and not by the individual action of each one [37]. 

There are many microbial genera used as starter cultures for fermented meat products. Although the most used belong to the group of lactic acid bacteria and Gram-positive catalase-positive cocci (GCC+), mainly represented by *Staphylococcus* spp. and *Kocuria* spp. [4], other starter cultures belong *Lactococcus* spp., *Leuconostoc* spp., *Enterococcocus* spp. and *Pediococcus* spp. are also used [13]. Moreover, yeast and moulds, that confer specific sensory characteristics, are also added as starter cultures. Starter yeast and moulds are mainly represented by *Debaromyces* spp. and *Aspergillus* spp., respectively. Moulds, since they are aerobic, are used as surface microbiota aimed to improve particular sensory and external characteristics. 

Regarding food safety, fermented meat products are considered as safe products due to the development of unfavourable or inhibitory conditions to the growth of spoilage and/or pathogenic microorganisms. Low values of pH and aW, presence of salt, nitrites, spices and other ingredients, called hurdle technology, are responsible for the pathogenic and spoilage microorganism inhibition [38]. But these hurdles, in some cases, are not enough, and foodborne pathogens can survive, causing outbreaks [39]. 

Thus, some industrial practices such as the reduction of fermentation times to increase the production yield, slicing, decreased salt content or decrease/absence of nitrites allow conditions for the survival of foodborne pathogens [40,41,42,43]. In addition, low initial natural microbial load of meat batter for fermented sausage manufacture may pose a risk for pathogen multiplication due to the reduced competition [44]. In this context, starter cultures present a key role in the guarantee of the safety of these products. Starter cultures are also used in combination with other techniques (e.g., essential oils, packaging) to improve its efficiency, guaranteeing the food safety [45]. 

### 2.1. Antimicrobial Effect of Selected Starter Cultures Against Foodborne Pathogens

LAB represent the main starter cultures used in the meat industry. Its antimicrobial effect has already been described decades ago, not only based on the reduction of the pH derived from the transformation of sugar into lactic acid but also by the competitive effect against natural microbiota, production of other organic acids (e.g., lactic acid, acetic acid, propionic acid, benzoic acid), hydrogen peroxide, enzymes or bactericidal peptides called bacteriocins [46], for which the action mechanism has been described elsewhere [4].

The antimicrobial effect of organic acids lies in the reduction of pH and in the action of undissociated acid molecules. Also, low pH facilitates the diffusion of organic acids across the cell membrane, collapsing the electrochemical proton gradient, affecting the cell membrane permeability and leading to the cell death [47]. Bacteriocins, most of them produced by LAB, are peptides or proteins of low molecular weight, synthesised in the ribosomes of the producer bacteria. Most bacteriocins act on the cellular membrane, destabilising and permeabilising through the formation of ionic channels or pores, which will release compounds such as phosphate, potassium, amino acids and adenosine triphosphate (ATP), decreasing the synthesis of macromolecules and consequently, cell death [48].

As previously discussed, starter cultures improve the safety of fermented meat products but evaluation of its antimicrobial effect, both in vitro and in food matrix, should be previously investigated. This study should be carried out both for commercial starters as well as in-house starters isolated from meat products or its environment of a specific meat industry [49]. This fact is important since less antimicrobial effect is usually described in real meat sausages than in in vitro assays related to the interaction with food compounds. Thus, Reference [16] verified that 1 out of 13 strains of *Latilactobacillus sakei* isolated from traditional meat sausages displayed an in vitro antimicrobial effect against *L. monocytogenes*, *Salmonella* spp. and *S. aureus*. Other research [50] observed that only 14 out of 39 commercial starter cultures for meat sausage manufacture displayed antimicrobial effect. In contrast, other authors [51] observed, both in broth and in fermented Greek sausage, that autochthonous strains of *Lb. sakei* displayed antimicrobial effect against *E. coli* and *L. monocytogenes*. Similar results were described [52] in meat model media and fermented sausage against *L. monocytogenes* using *Enterococcus mundtii* as a starter culture. However, differences observed in the antimicrobial effect of starter cultures can be related to the microorganism, strain, the target microorganism and/or characteristics of sausage manufacture [53]. Thus, it was observed [54] that addition of *Lacticasebacillus rhamnosus* as a starter culture, isolated from human intestinal tract, did not suppress the growth of enterotoxin-producing *S. aureus*. 

Antimicrobial effect of meat-borne LAB has been described in the literature against main foodborne pathogens and main spoilage bacteria (Table 1). The antimicrobial effect is characterised by reducing or eliminating pathogenic and/or spoilage microorganisms in a shorter time during the manufacturing process. Thus, it allows meat producers to obtain safer products more quickly, being able to optimise the production processes. It is important to remark that the ability of starter cultures to compete with the natural microbiota of the raw material and to undertake the metabolic activities expected is conditioned by its growth rate and survival in the conditions prevailing in the fermented sausage (i.e., anaerobic atmosphere, NaCl concentration, ingredients, temperature of fermentation and ripening and low pH) [55]. 

Thus, technological agents such as salt and curing agent may interfere in the bacteriocin production of *Lb. sakei* [56]. Also, spices seem to influence the growth of starter cultures. Thus, it was observed [57] that garlic enhanced bacteriocin production, lactic acid production was stimulated by pepper, while nutmeg decreased the bacteriocin production. In contrast, addition of garlic in Turkish soudjuk manufacture did not present any significant effect on the survival of *S. typhimurium* [58].

In addition, in case of high microbial contamination, the antimicrobial effect of starter cultures can be compromised. For example, if the initial contamination level is high, the use of a starter culture cannot improve the quality of the food product [44]. Thus, it has been reported [59] that the antimicrobial effect of natural microbiota cannot be enough in high microbial contamination of meat batter of Italian salami with 7 log cfu/g of *Salmonella* spp. and *L. monocytogenes*. Although *Salmonella* spp. decreased about 4 log cfu/g after fermentation, *L. monocytogenes* reduced less than 1 log cfu/g. 

Also, the way in which starter cultures are added to the meat batter may influence its antimicrobial effect. Thus, microencapsulation of *Limosillactobacillus reuteri* decreased its antimicrobial effect against *E. coli* O157:H7 in dry fermented sausages [60].

Use of starter cultures combined with other compounds (Table 2), such as essential oils, organic acids, wine or spices, have been added to meat batter to improve the safety of these products [61,62,63,64,65,66]. However, previous assessment on potential interaction with starter cultures must be addressed since an inhibitory effect may be present, as discussed above. 

### 2.2. Control of Biogenic Amine Formation in Meat Products by Addition of Selected Starter Cultures

Biogenic amines (BA) are nitrogenous compounds that are found in fermented foods and beverages formed by the microbial decarboxylation of amino acids [67]. The main BAs in foods are histamine, tyramine, putrescine, cadaverine, tryptamine, spermine and spermidine. In some cases, they have been considered hazardous substances due to their ability to react with nitrites and form potentially carcinogenic nitrosamines [68].

Regarding consumers’ health, ingestion of BA may display some adverse dose-dependent effect, from allergy symptoms (e.g., skin rash, hives, itching) to systemic clinical signs (e.g., difficulty breathing, diarrhoea, vomiting, abdominal pain, joint pain, fatigue, seasickness, among others) [69]. In addition, due to the fact that BA are thermostable, further processing of foods will not eliminate them once formed [70]. 

Since concentration of BA in foods can display negative effects on the health of consumers, research about application of some manufacturing techniques and/or procedures, such as use of high hydrostatic pressure, control of NaCl concentration, freezing of raw materials, use of starter cultures, seasoning mixtures, product diameter, reduction in the amount of sugar added, use of additives, variation of the time/temperature parameters during fermentation and ripening, among others, have been investigated to decrease the BA contents in final product [84,85,86,87,88,89,90,91]. The microbiological quality of meat sausage ingredients is related to the aminogenesis process. Although hygienic quality is essential, other technological measures are needed. Thus, use of starter cultures represents one of the main measures to control BA formation [87]. The action mechanism of starter cultures is based on its competitive effect against the natural microbiota. Several studies have demonstrated the role of starter cultures in reducing the accumulation of BA in meat products. For example, combination of *S. xylosus* and *Lactiplantibacillus plantarum* decreases the content of cadaverine, putrescine, tryptamine, 2-phenylethylamine, histamine and tyramine by about 50% in Chinese Harbin dry sausage [92]. Addition of *Lb. plantarum* decreases the BA content by about 20%, but addition of both of them displayed a synergistic effect in which starter mix reduced tryptamine, phenylethylamine, putrescine, cadaverine, histamine and tyramine contents by nearly 100%, 100%, 86%, 63%, 82% and 43%, respectively [93].

Other research indicated that the combination of *Enterococcus thailandicus* and *Enterococcus faecalis* displayed better antibiogenic formation than the combination of *Staphylococcus carnosus* and *Lb. sakei* [94]. Since pH affects the BA formation, the lower level of pH achieved by the combination of *E. thailandicus*/*E. faecalis* than those achieved by the combination of *S. carnosus/Lb. sakei* may explain this difference on the anti-biogenic properties.

In contrast, addition of starter *Lb. sakei* and *S. xylosus* in the manufacture of Italian sausages displayed a higher level of BA compared to those made without starters [95]. This result can be explained by the aminobiogenic capacity of both starter cultures in which histidine [96] and tyramine [97] decarboxilase activity was reported in artisanal fermented sausages. This fact was also reported in foal dry sausage, in which the use of a mix of *Pediococcus pentosaceus* and *S. xylosus* displayed higher accumulation of BA than those made without starter [98]. Combination of *Staphylococcus equorum* S2M7/*Lb. sakei* CV3C2 displayed better anti-biogenic performance than *S. xylosus* CECT7057/*Lb. sakei* CECT7056 in finished dry-cured sausage Paio Alentejano. However, addition of yeast 2RB4 together *S. equorum* S2M7/*Lb. sakei* CV3C2 reduced the BA content in the finished product by about 10%. The yeast effect may probably be associated to an improved competitive effect against other naturally bacterial strains presented in dry-cured sausage able to produce biogenic amines [99].

As indicated above, starter cultures may prevent the BA formation by its competitive effect against spoilage bacteria, however, recent research reported that LAB have been considered as main BA producers [68]. It indicates that selected starter cultures used in fermented sausage manufacture must be previously assessed regarding their decarboxilase activity. Since starters used in fermented meat products are usually isolated from natural microbiota, aminobiogenic capacity vary among LAB [100]. Thus, it has been reported that 80% of indigenous *E. faecium* and *E. faecalis* presented tyramine-producing capacity [101,102]. However, the combination of *E. thailandicus* and *E. faecalis* produced the lowest BA concentration [94]. With regards to *S. xylosus*, only 7 out of 50 strains isolated from artisanal Italian sausages presented potential capacity to produce spermine, spermidine, tryptamine or tyramine [97]. Regarding *Lb. sakei*, this LAB has been reported as non-aminobiogenic [100] and may explain why manufacture of Italian sausage with a mix of *Lb. sakei* and *S. xylosus* displayed lower BA content than addition of a starter mix composed of *P. pentosaceus* and *S. xylosus* [103]. 

Overall, the aminobiogenic capacity of LAB together with the BA capacity of spoilage microbiota (naturally presented in raw meat) represent a chemical hazard concern in fermented meat products. It highlights the importance of selecting strains with oxidase activity instead of decarboxilase activity as starter cultures [104]. Although production of BA during sausage manufacture is inevitable, rapid overgrowth of selected (non-aminobiogenic starter) LAB at the beginning of fermentation may improve the chemical safety of these products. 

Nitrates and nitrites in cured meat products are responsible for the characteristic red colour, inhibit the growth of pathogenic bacteria such as *Clostridium botulinum*, contribute to the development of the typical aroma of cured meats and act as antioxidants by delaying the development of rancidity and avoiding the appearance of alterations [105].

Overall, nitrates are not toxic, except in case of ingestion of large amounts. However, nitrites may pose a risk derived from their consumption since they can lead to allergic reactions and even cause methemoglobinemia situations. The main concern of nitrites is related to the possibility to act as precursors in the formation of carcinogenic nitrosamines, both in foods and at the organic level (for example, under acidic pH of mouth or stomach, nitrites or nitrates added to food or naturally occurring may combine with amines to form nitrosamines) [106]. Nitrosamine is a general term used to designate a vast group of *N*-nitroso compounds (NOCs). Its importance relies on the evidence of their carcinogenic properties [107]. Specifically, nitrosamines are formed by the reaction of compounds derived from nitrites, such as nitrous acid, with secondary amines throughout a nitrosation reaction. The presence of amines and the addition of nitrates and nitrites during the preparation of cured meat products can favour the development of this type of reaction in them. In meat products, the most commonly detected volatile nitrosamines are N-nitrosodimethylamine (NDMA), N-nitrosopyrrolidine (NPYR), N-nitrosopiperidine (NPIP), N-nitrosodiethylamine (NDEA), N- nitrosodi-n-butylamine (NDBA) and N-nitrosomor-folin (NMOR) [40]. Regarding these health issues for consumers, several food processing techniques have been investigated to reduce or replace the use of nitrates and nitrites in meat products (i.e., irradiation and n-nitrosamine blockers such as ascorbic acid) [106].

In this context, starter cultures appear to have a role in the reduction of nitrite levels in cured meat products. Thus, some authors [108,109,110] reported that addition of *L. plantarum*, *L. pentosus*, *Lb. sakei* or *Lb. curvatus* as starter culture decreased the nitrite content, suggesting the existence of nitrite reductase and heme-independent nitrite reductase that converts nitrite to NO, NO_2_ or N_2_O under anaerobic conditions [111]. Also, it has been referred that the rate of nitrite dissipation increases with pH reduction [112]. In contrast, the use of starter culture increased the N-nitrosopiperidine levels in heat-treated Turkish sucuk [113].

### 2.3. Control of Polycyclic Aromatic Hydrocarbons in Meat Products by Addition of Selected Starter Cultures

Polycyclic aromatic hydrocarbons (PAH) constitute a large group of organic compounds widely distributed in the environment with carcinogenic effects. Food contamination can occur by atmospheric deposition processes as well as during processing mainly related to heat treatments such as smoking, either by traditional methods or by the addition of smoke extracts directly into foods by spraying or dipping [114].

Since PAHs represent a health hazard and meat and meat products are one of the food categories contributing most to the dietary PAHs intake per day of the European Union, maximum levels in foods have been set by specific policy [115] to reduce its exposition. Research about the influence of starter cultures on PAH reduction is scarce.

Recently, it has been reported that immersion of cold smoked pork sausages in a LAB suspension of *Lb. sakei*, *P. acidilactici* and *P. pentosaceus* before ripening or in finished products decreased the benzo[a]pyrene contents [116]. Although the action mechanism of PAHs’ reduction is still unknown, it has been suggested that toxins are removed by specific enzymes produced by cells [117]. However, other studies suggested that biodegradation may be related to PAH binding to wall components of LAB cells [118]. Also, binding mechanisms of ion-exchange and hydrophobic bonds between exopolysaccharides and PAH have been suggested as a biodegradation route [119].

However, the effect of commercial starter (*Lactobacillus* spp., *Micrococcaceae* and yeasts) vs experimental starter (*Lb. sakei* and *S. xylosus*) on the PAH content in finished Portuguese Paio Alentejano (dry-cured pork sausage) did not evidence significant differences among starters [120]. It may suggest that the presence of specific enzymes or the presence of specific membrane compounds, as previously indicated, can be associated to specific microorganisms and/or strains.

## 3. Use of Starter Cultures to Improve the Safety in Dairy Products

### 3.1. Improving the Food Safety of Cheese by Use of Starter Cultures

LAB are the main starter cultures used in the dairy industry for cheese and yogurt production. Most of them are grouped into the genera *Lactococcus*, *Lactobacillus*, *Leuconostoc* and *Pediococcus*. Along with LAB, species of other genera such as *Propionibacterium* and *Bifidobacterium* are also occasionally used.

As previously discussed, use of starter cultures allows manufacturers to control and optimise the fermentation processes aimed to confer specific characteristics to the final product. Thus, starter cultures are related to the flavour and aroma characteristics, proteolytic and lipolytic activities, as well as inhibition of pathogenic microorganisms. In this section, we discuss the use of starter cultures to improve the safety of cheese and yogurt.

In cheese manufacturing, lactic acid bacteria play different roles in the cheese making process. Some species participate more in fermentation while others are mainly involved in ripening. Regarding food safety, the importance of LAB is related to the antimicrobial effect against foodborne and spoilage bacteria throughout production of organic acids, competitive effect and production of antimicrobial substances [25].

Regarding foodborne pathogens, the most commonly involved in cheese outbreaks are enteropathogenic *E. coli*, particularly 0157:H7, *Salmonella* spp., *S. aureus* and *L. monocytogenes* [1]. This last microorganism represents the most concerning pathogen since they can survive in a wide range of conditions during manufacture, ripening and storage (even in chilled storage). 

Use of starter cultures to control *L. monocytogenes* in cheese has been largely described in the literature (Table 3), mainly based on the bacteriocinogenic properties of starter cultures. Thus, use of bacteriocinogenic starter cultures of sakacin, nisin, pediocin or enterocin represents the most important tool to control *L. monocytogenes* in cheese [121,122]. However, control of surface contamination by *L. monocytogenes* by LAB, during ripening or storage, should be carefully assessed since susceptibility of *Listeria* spp. to the antimicrobial activity of LAB is strain-dependent [123]. This strain susceptibility has been reported by other authors [122], in which addition of starter *Lactococcus lactis* in fresh cheese displayed a modest decrease of *L. monocytogenes* counts. The authors of Reference [124] reported that spraying surfaces with *E. faecium* in Munster cheese did not decrease *L. monocytogenes* levels but acts as a bacteriostatic. It can be concluded that starter cultures play an important role in the control of *L. monocytogenes,* but antimicrobial properties should be previously assessed in vitro (as described above for meat products) since *L. monocytogenes* susceptibility is strain-dependent. This fact was reported in Reference [125], in which nearly one third out of eight hundred LAB strains displayed anti-listerial activity. Also, hygienic practices must be guaranteed since this pathogen and outbreaks are still detected [126,127]. To improve the safety of cheese, combination of starter cultures and other antimicrobial treatments have been studied. Thus, enhancing the anti-listerial effect of starter *Lc. lactis* with lactic acid and sodium lactate [128] or plus sodium acetate or sodium lactate [122] have been reported. Addition of tartaric, fumaric, lactic or malic acid improves the inhibition of *L. monocytogenes* [129], but differences among organic acids may be explained by differences in the synergistic effect with lactic or acetic acid naturally produced by LAB during cheese ripening.

Combination of essential oils and starter cultures during cheese manufacture should be assessed since survival of starter can be compromised with further impacts on sensory and safety characteristics [130,131].

*S. aureus* is a concerning pathogen in cheese making. The importance of its control is related to the capacity of toxin production that, once formed in food, are extremely difficult to eliminate. These toxins are responsible for most staphylococcal food poisoning associated with the consumption of contaminated food. Thus, control of *S. aureus* contamination is of great importance, with special relevance in those cheeses made from raw milk since prevalence of *S. aureus* in milk is high [132]. Indeed, microbiological criteria for *S. aureus* in cheese has been set by law [133].

Research about control of *S. aureus* by addition of starter cultures is less than observed for *L. monocytogenes* (Table 3). Reduction of *S. aureus* was only achieved in bacteriocin producer *Lc. lactis* strains [134]. However, other reports suggest that the presence of *S. aureus* in raw milk is inhibited at different stages of ripening [135]. It has been observed that *S. aureus* survives in 60-day ripened white cheese made with commercial starter, although combination with probiotic [136] *L. rhamnosus* and *Lactobacillus casei Shirota* displayed an inhibitory effect up to 5 Log cfu/g, probably associated with the increased effect of bacteriocins arising during the ripening period. In contrast, use of starter *L. rhamnosus* did not display an inhibitory effect against *S. aureus* in Brazilian minas frescal cheese [137]. Survival of *S. aureus* in Jben, a Moroccan fresh cheese, was also reported [138], but addition of nisin-producer starter *Lactococcus lactis* subsp. *lactis* UL730 increased the safety of fresh cheese by elimination of *S. aureus* after 4 days. Combination of *Lc. lactis* subsp. *cremoris* and oregano essential oil (EO) to inhibit *L. monocytogenes* and *S. aureus* has been studied [131], however its efficacy may be compromised due to the inhibitory effect of oregano EO against added starter culture. A similar inhibition effect of EO was observed in a combination of thymus EO and starter *Lc. lactis* subsp. *lactis* and *Lc. lactis* subsp. *cremoris* against *S. aureus* [130]. Combination of *Mentha longifolia* L. EO in combination with starter *Lb. casei* in concentrations over 50 ppm displayed a synergistic effect against growth of *S. aureus* [139]. In addition to the negative effect of EO on starter LABs, as described above, sensory cheese analysis is necessary since inhibitory concentrations of EO may be incompatible with consumer acceptance.

Application of high-pressure treatment (HPT) at lower pressure in combination with bacteriocin-producing LAB [140] improves the safety of raw cheese against *S. aureus*. Since HPT disrupts the structure of *S. aureus*, including its cell membrane, it may explain the enhanced effect of bacteriocins produced by starter LAB.

In cheese processing, *Salmonella* spp. decreases along the ripening and storage periods [141,142]. Factors such as salt concentration, storage temperature and pH are the main barriers that disrupt its growth. However, *Salmonella* spp. may survive until the finished product [143,144]. Thus, it has been suggested that reduction of *S. typhymurium* along ripening in Montasio cheese is associated with the drop of pH after the negative antagonistic effect of starter *Lb. plantarum* by the spot method [145]. Survival of *Salmonella* spp. in low-salt cheddar cheese made with commercial starter *Lactococcus lactis*, *Lc. lactis* subsp. *cremoris* and *Lb. helveticus* was detected for up to 90 days when stored at 4 or 10 °C and for up to 30 days at 21 °C. Addition of starter cultures in cheese making improved the decrease of *Salmonella* spp. [146,147], probably associated to the enhanced effect of the pH by lactic acid production [148]. However, the survival of this pathogen indicates that the antimicrobial effect of starter cultures used in cheese making must be previously verified together with high hygienic quality of ingredients and storage temperature conditions.

Presence of *E. coli* in cheese has been reported in the literature. During cheese making, *E. coli* increased in the first hours of ripening [157,158]. Thus, the use of starter cultures to inhibit *E. coli* growth has been investigated as a biopreservative tool [157]. Addition of nisin- and pediocin-producing *Lc. lactis* CL2 inhibited *E. coli* after 15 and 30 days of ripening. However, addition of non-bacteriocinogenic *Lc. lactis* ESI 153 [121] displayed an unexpected better inhibitory effect than pediocin-producer *P. acidilactici*. 

It was also reported that the inhibitory effect of starter cultures (*Hafnia alvei, Lb. plantarum* and *Lc. Lactis)* against *E. coli* may be influenced by the initial LAB load of raw milk [159]. It suggests that the acidification rate carried out by natural LAB microbiota together with starter cultures is related to the inhibitory effect of *E. coli*. However, it has been suggested that survival of *E. coli* during ripening may be associated to the initial microbial load of raw milk [160]. The synergistic effect of essential oil and starter cultures to control *E. coli* have been also studied [161], in which the combination of *Zataria multiflora* EO and *Lb. acidophilus* decreased the growth rate of *E coli*. In contrast, total growth inhibition of *E. coli* was achieved by combination of *Lb. acidophilus* LA-5 with oregano and rosemary EO [162].

Combination of bacteriocinogenic starter cultures and high hydrostatic pressure can reduce *E. coli* counts with lower pressure intensity in ripened cheese [121]. Other authors showed that addition of *Lb. reuteri* or glycerol in semi-hard cheese manufacture does not inhibit the growth of *E. coli* O157:H7 up to 30 days of ripening. However, combination of *Lb. reuteri* and glycerol eliminates *E. coli* completely after 24 h [163].

### 3.2. Improving the Food Safety of Yogurt by Use of Starter Cultures

Yogurt is a food product obtained by lactic fermentation of milk previously subjected to a heat treatment, at least, to pasteurisation, through the action of some microorganisms such as *Lactobacillus delbrueckii* subsp. *bulgaricus* and *Streptococcus thermophilus*. Also, other lactobacilli and bifidobacteria are sometimes added during or after culturing yogurt as probiotics [164]. Yogurt is considered a safe food since its manufacture includes two hurdle steps that make the survival of foodborne pathogens difficult, such as heated milk and low pH resulting from fermentation. To the best knowledge of the authors, no information regarding recent bacterial outbreaks in yogurt is available, although older scientific studies have reported the presence of foodborne pathogens such as *L. monocytogenes*, *E. coli* or *Yersinia enterocolitica* [165,166] related to cross-contamination issues.

Because yogurt manufacture is carried out by addition of *Lactobacillus delbrueckii subsp. bulgaricus* and *Streptococcus thermophilus* starters, scarce research is available regarding the role of other starter cultures added to improve its safety. 

Addition of bacteriocinogenic *Streptococcus thermophilus* as a starter displayed an inhibitory effect against *L. monocytogenes* during fermentation [167]. However, a scarce inhibitory effect was observed against *S. aureus* by the same starter. Counts of *L. monocytogenes* and *E. coli* increased during fermentation but decreased during storage, influenced by storage temperature, with a higher decrease at 10 than at 4 °C [168]. In addition, fermentation at two consecutive periods (43 °C for 3 h and 30 °C for 21 h) revealed better inhibition effect against *E. coli* O157:H7, *L. monocytogenes* 4b and *Y. enterocolitica O3* [165]. Greater inhibition results against *E. coli* were also observed at 17 and 22 °C than at 4 and 8 °C during yogurt storage, suggesting that *E. coli* presented more adaptation capacity to pH variation than refrigeration temperatures [169]. 

Regarding *Salmonella* spp., it was observed that *S. enteritidis* and *S. typhimurium* survived throughout the fermentation process [170]. Also, *S. enteritidis* can survive up to 12 days at 4 °C and up to 60 h at 25 °C [170]. Overall, Gram-negative bacteria discussed above presented variable capabilities of survival throughout fermentation and storage of yogurt [171]. So, contamination after fermentation may represent a risk for foodborne poisoning. This survival on acid conditions can be related to development of acid, gene-encoded survival mechanisms [172]. It indicates that safety of yogurt cannot be based on the antimicrobial effect of starter cultures added. Since one of the antimicrobial effects of starters is based on the competitive effect, the survival mechanisms of enterobacteriaceae at acid environment may overlap the growth capacity of the starter. Also, the fact that not all starter cultures presented bacteriocinogenic capacity may imply a previous testing, as already discussed in the text. In consequence, good microbial quality of milk, proper thermal treatment of milk, together with good hygienic manufacturing practices and proper starter cultures selection must be implemented [173]. 

## 4. Use of Starter Cultures to Improve the Safety of Fish Products

Fermented fish products are part of the daily dietary habits in some regions of Asia and are considered as healthy foods since fermentation results not only in a shelf-life extension but also results in the production of probiotic metabolites. Since fermented fish products are made from fresh fish, they can be easily altered by spoilage and pathogenic bacteria and also may accumulate toxins such as histamine and other BA in excessive concentrations. Manufacture of fermented fish does not include steps such as cooking or pasteurisation which eliminate foodborne pathogens. Thus, microbiological safety of fermented fish products therefore depends on rapid and sufficient fermentation by lactic acid bacteria. Regarding microbiological quality, foodborne pathogens such as *E. coli*, *S. aureus* or *Vibrio cholerae* have been reported in the literature [174]. It is important to highlight that most fermented fish products are often made from fish with low market value (in an attempt to decrease fish waste) and processed in the artisanal fish-processing industry, in which facilities and hygienic processing may pose a risk of foodborne illness [175]. Microbes involved in fish fermentation include lactic acid bacteria such as *Lactobacillus*, *Leuconostoc*, *Micrococcus* or *Pediococcus,* as well as other microorganisms such as *Streptococcus*, *Enterobacter*, *Pseudomonas*, *Bacillus* spp. or *Proteus* spp. [176].

### 4.1. Antimicrobial Effect of Selected Starter Cultures Against Foodborne Pathogens in Fish Products

Fish microbiota (mucus, internal organs and gill) are related to the surrounding environment and are responsible for the post-mortem changes (sensory, autolytic and bacteriological) after fishing. These biochemical processes presented some importance since use of low-quality raw fish to be processed into fermented products is a common practice in an attempt to save deteriorated fish. Although use of low-quality raw fish for fermented fish manufacture reduces food waste, this practice represents a risk for foodborne intoxication [175]. Thus, use of starter cultures represents an important tool to increase the safety of these products. For example, addition of a mix of *Lb. plantarum* IFRPD P15 and *Lb. reuteri* IFRPD P1 displayed a synergic effect against *E. coli* in contaminated plaa soom thai fermented fish, being undetectable after 24 h [177]. However, in batches made only with *Lb. plantarum* IFRPD P15, *Salmonella* spp. was detected until 48 h, suggesting that the inhibitory effect of LAB against *Samonella* spp. may depend on the starter microbial species. Thus, it was reported that *S. enterica* serovar *weltevrede* may survive during the fermentation process in Thai som-fak, a low-salt garlic Thai fermented fish [178]. It is important to remark that microbiological safety is associated with a successful fermentation achieved by rapid growth and acid production of the natural LAB microbiota naturally present in fish. Since carbohydrate content in fish is very low, fermentable substrate must be added, with rice being the most often used. Thus, to improve the safety regarding foodborne pathogens, addition of a mix of starter cultures more efficiently decreased foodborne and spoilage bacteria [179,180].

Seafood and fishery products are perishable foods related to the rapid microbiological spoilage. In an attempt to extend its shelf-life, some researchers have evaluated the use of LAB as bioprotective cultures [181,182]. Thus, in non-fermented fish products, starter sakacin producers *Lb. sakei* CTC494, *Lb. curvatus* CTC1741 and *Carnobacterium maltaromaticum* have been used as bioprotective cultures to control L. monocytogenes in chilled smoked salmon with different anti-listerial extents [183]. Similar anti-listerial effects were observed in modified atmosphere packaging of filleted gilthead sea bream by addition of the same *Lb. sakei* CTC494 starter [184], as well as in vacuum-packaged cold-smoked salmon by addition of *E. faecium* ET05 [185]. Shelf-life extension using LAB from marine origin was reported [181]. Thus, use of *Lactobacillus curvatus* BCS35 and *Enterococcus faecium* BNM58, previously isolated from fish and fish products, extends the shelf-life of young hake (*Merluccius merluccius*) and megrim (*Lepidorhombus boscii),* also inhibiting L. monocytogenes. Shelf-life extension and inhibition of L. monocytogenes in vacuum-packed Salmon (Salmo salar) was also studied [182] by addition of selected LAB (*Carnobacterium maltaromaticum*, *Lactococcus piscium*, *Leuconostoc gelidum*, *Vagococcus fluvialis*, *Carnobacterium inhibens*, *Aerococcus viridans*) isolated from fishery products. Although LAB improved the shelf-life and inhibited *L. monocytogenes*, its competitive effect against natural microbiota differed among the starter LAB used, highlighting the need of proper previous assessment on the selection of LAB as bioprotective cultures.

Dipping fresh tilapia fillets for sashimi in a suspension of *Lb. plantarum* 1.19 modified the spoilage microbiota. Although *Pseudomonads* and *Aeromonas* were scarcely inhibited, *Lb. plantarum* 1.19 inhibited the growth of *Micrococcus* spp., contributing to an improvement of shelf-life [186]. Similar results on shelf-life extension were observed in ribbonfish treated with *Lactobacillus plantarum* SKD4 and *Pediococcus stilesii* SKD11 [187].

### 4.2. Control of Biogenic Amine Formation by Addition of Selected Starter Cultures in Fish Products

During fish fermentation, BA values increase during the first days of fermentation associated to the amino acid decarboxilase activity of natural microbiota present in the slime of the body, guts and gills [179,188].

Histamine represents the main chemical hazard, frequently associated with health problems after fish consumption. Moreover, its potential toxicity can be enhanced by other BA, such as putrescine, cadaverine or tyramine. Some foodborne pathogens also have the ability to produce BA due to histidine decarboxylase activity [189]. Thus, hygienic quality of fish may influence the content of BA during fermentation. As previously discussed for fermented meat products, use of starter cultures represents one of the main measures to control BA formation based on its competitive effect against the natural microbiota.

Addition of *Lb. plantarum* 120, *Saccharomyces cerevisiae* 2018 and *S. xylosus* as starters decreased the N-nitrosodimethylamine and its precursors to different extents in low-salt Chinese traditional fermented fish [190]. Different inhibition effect on BA formation depends on the starter species used [179], in which addition of *Lb. plantarum* KM1450 was more efficient in reducing BA accumulation than *Lb. sakei* KM5474 during som-fug fermentation [191]. Also, addition of starter mix (*Lb. plantarum*/*S. xylosus*/*P. pentosaceus* ATCC3331, *Lb. plantarum*/*S. xylosus*/*Lb. casei* subsp. *casei* - *S. xylosus*/*Lb. casei* subsp. *casei*/*P. pentosaceus* ATCC3331) decreased histamine concentration by about 90%; however, tyramine, putrescine and cadaverine increased, but in lower concentrations than batches with no starter cultures [179]. A similar synergistic effect in decreasing the BA formation was described in bighead carp surimi, in which addition of *Lb. casei* 6002, *Streptococcus lactis* 6018, *S. cerevisiae* Hansen-1049, and *Monascus anka*-5037 reduced on average 65% of histamine, tyramidine, spermidine and spermine content after 24 h of fermentation [192]. However, a slight increase, about 8%, was observed for putrescine in batches made with starter, added in accordance to that reported in Reference [179].

Combinations of different mixes of starter cultures, (1) *Lb. plantarum* 120, *S. xylosus* 135 and *S. cerevisiae* 31 (1:1:1), or (2) *P. pentosaceus* 220, *S. xylosus* 135 and *S. cerevisiae* 22 (1:1:1), improved the control of tyramine, putrescine, cadaverine and spermidine formation in suan yu fermented fish [193]. Combination of starter cultures seems to have a greater inhibition effect on the formation of BA, probably associated with the rapid decrease of pH that affects the growth of *Enterobacteriaceae* and *Pseudomonas*, the main spoilage bacteria of fish [180,192]. It is important to remark that starter cultures for fish fermentation must present oxidase activity instead of decarboxylase activity (aminobiogenic activity), as previously discussed. 

## 5. Conclusions

Starter cultures can be defined as preparations with a large number of cells that include a single type or a mixture of two or more microorganisms that are added to foods in order to take advantage of the compounds or products derived from their metabolism or enzymatic activity. Production of fermented foods is, in most cases, based on traditional recipes, indicating that natural and uncontrolled food environment conditions may affect the final characteristics of food. Regarding food safety, starter cultures inhibit the growth of foodborne and spoilage bacteria mainly based on the acid production and subsequent drop of pH. However, production of other organic acids as well as antimicrobial substances (bacteriocins) suggest that the inhibitory effect is due to a more complex antagonistic system. As previously discussed, several studies evidenced the antagonistic effect of starter cultures against main foodborne pathogens in food from animal origin. Moreover, starter cultures also play a relevant role in the control of chemical hazards such as BA or PAH, although more research its necessary to evidence the inhibitory mechanisms. It can be concluded that use of starter cultures represents a natural alternative to guarantee food safety in a context in which consumers are looking for less processed and more natural foods. However, some considerations are necessary to take into account. Since most of the starter cultures used are isolated from specific (traditional) products and/or from their production environments, it implies that the potential antagonistic effect against foodborne and spoilage bacteria as well its inhibition effect on BA and/or PAH contents must be previously assessed both in vitro and in real food products. Thus, selection of a starter culture should be carried out within the scope of its application because its function will depend on the type of fermented food, the technology applied, the ripening conditions, raw materials and other ingredients.

## Figures and Tables

**Table 1:** Antimicrobial effect of selected starter cultures (added as ingredients during fermented meat manufacture) against main foodborne pathogens.

Starter(s) Culture(s) Used	Origin of the Starter Culture	Characterisation of the Inhibition Mechanism	Reference
(a) Starter cultures selected against *L. innocua*			
*Pediococcus acidilactici*	Isolated from alheira (Portuguese fermented pork sausage)	Not determined	[71]
(b) Starter cultures selected against *L. monocytogenes*			
*Lactiplantibacillus plantarum* (strain 178) *(formerly Lactobacillus plantarum)*	Isolated from pork meat	Not determined	[72]
*Lactiplantibacillus plantarum*	Isolated from poto-poto, an ethnic maize fermented food	Production of plantaricin	[73]
*Latilactobacillus sakei* (formerly *Lactobacillus sakei)*	Isolated from chouriço (fermented cured pork sausage) made from wine-marinated meat	Not determined	[16]
*Latilactobacillus curvatus* 54M16 (formerly *Lactobacillus curvatus*)	isolated from traditional fermented sausages of Campania region (Italy)	Bacteriocing genes detection by PCR	[74]
*Pediococcus pentosaceus*	IOTEC culture collection (Thailan)	Not determined	[75]
Mix of *Staphylococcus xylosus* DD-34, *Pediococcus acidilactici* PA-2, *Lactobacillus bavaricus* MI-401	Commercial starter culures (FloraCarn LC, Mœller RM 52)	Production of pediocin (indicated by manufacturer)	[76]
*Pediococcus acidilactici*	Commercial stater cultures from Chr. HansenLaboratories (Denmark)	Bacteriocin purification and amino acid sequencing	[50]
*Latilactobaciullus sakei 8416* *Latilactobacilus sakei 4413*	Natural Greek dry-fermented sausage	Not determined	[51]
*Lacticaseibacillus rhamnosus E-97800 (formely Lactobacillus rhamnosus)* E-97800; *Lacticaseibacillus rhamnosus* LC-705; *Lactiplantibacillus* *plantarum* ALC01; *Pediococcus pentosaceus* RM2000	*Lacticaseibacillusrhamnosus* E-97800: isolated from human faeces; *Lacticaseibacillus* *rhamnosus* LC-705: isolated from dairy; *Lactiplantibacillus plantarum* ALC01: commercial starter *Pediococcus pentosaceus* RM2000: commercial starter	Not determined	[77]
(c) Starter cultures selected against *Clostridium perfringens*			
*Lactiplantibacillus plantarum PCS20*	Microbial Strain Collection of Latvia,	Not determined	[60]
*Pediococcus acidilactici*	Commercial stater cultures from Chr. HansenLaboratories (Denmark)	Bacteriocin purification and amino acid sequencing	[50]
(d) Starter cultures selected against *Salmonella* spp.			
*Enterococcus faecalis* (strains A-48-32 and S-32-81)	Isolated from cheese	Production of enterocin	[78]
*Latilactobaciullus sakei*	Isolated from chouriço (fermented cured pork sausage) made from wine-marinated meat	Not determined	[16]
*Latilactobacillus sakei* 23K *Latilactobacillus sakei* BMG 95 *Latilactobacillus sakei* BMG 37 *Staphylococcus xylosus*	*Latilactobacillussakei* 23K: isolated from a French sausage *Latilactobacillus* *sakei* BMG 95: isolated from anchovies ‘*Latilactobacillus sakei* BMG 37: isolated from sheep meat *Staphylococcus xylosus*: isolated from artisanal Tunisian fermented sausages	Not determined	[79]
(e) Starter cultures selected against *Escherichia coli*			
*Lactiplantibacillus plantarum* (strain 178)	Isolated from pork meat	Not determined	[72]
*Lactiplantibacillus plantarum*	Isolated from poto-poto, an ethnic maize fermented food	Production of plantaricin	[73]
*Lacticaseibacillus rhamnosus* (strains GG, E-97800 and LC-705) and *Pediococcus pentosaceus*	*Lacticaseibacillus rhamnosus* (strains GG, LC-705): commercial starter (Valio Ltd., Helsinki, Finland) *Lacticaseibacillus rhamnosus* E-97800: commercial starter (VTT Biotechnology, Finland) *Pediococcus pentosaceus:* commercial (Gewurzmuller, Germany)	Not determined	[80]
*Latilactobaciullus sakei* *Leuconostoc mesenteroides*	Fermented game meat sausages	Not determined	[81]
*Limosilactobacillus reuteri* ATCC 55730 (formerly Lactobacillus reuteri)	American Type Culture Collection	Production of reuterin	[60]
*Latilactobaciullus sakei 8416* *Latilactobacilus sakei 4413*	Natural Greek dry-fermented sausage	Not determined	[51]
(f) Starter cultures selected against *Staphylococcus aureus*			
*Enterococcus faecalis*	Isolated from cheese	Production of enterocin	[78]
*Lactiplantibacillus plantarum* (strain 178)	Isolated from pork meat	Not determined	[72]
*Latilactobaciullus sakei*	Isolated from chouriço (fermented cured pork sausage) made from wine-marinated meat	Not determined	[16]
*Latilactobacillus sakei* 23K *Latilactobacillus sakei* BMG 95 *Latilactobacillus sakei* BMG 37 *Staphylococcus xylosus*	*Latilactobacillussakei* 23K: isolated from a French sausage *Latilactobacillus* *sakei* BMG 95: isolated from anchovies *Latilactobacillus* *sakei* BMG 37: isolated from sheep meat *Staphylococcus xylosus*: isolated from artisanal Tunisian fermented sausages	Not determined	[79]
*Lacticaseibacillus rhamnosus* FERM P-15120 *Lacticaseibacillus paracasei* subsp. *paracasei* FERM P-15121 (formerly *Lactobacillus paracasei*)	Isolated from intestinal tracts	Not determined	[54]
(g) Starter cultures selected against *Enterobacteriaceae*			
Mix of *Pediococcus acidilactici* (MC184, MS198 and MS200) plus *Staphylococcus vitulus* RS34	Isolated from traditional Iberian dry-fermented salchichón	Not determined	[61]
(h) Starter cultures selected against *Yersinia enterocolitica*			
*Latilactobacillus sakei* ATCC 15521 *Pediococcus acidilactici*	*Latilactobacillus sakei* ATCC 15521: obtained from the American Type Culture Collection *Pediococcus acidilactici*: obtained from the Food Microbiology Culture Collection (Kansas State University, Manhattan, Kan., USA)	Not determined	[82]

PCR: polymerase chain reaction. The nomenclature of the genus *Lactobacillus* was presented according to the new taxonomic classification [83].

**Table 2:** Combination of starter cultures and other hurdle technology to improve antimicrobial effect against foodborne pathogens in meat products’ manufacture.

Starter Culture	Origin of Starter Cultures	Combined by	Antimicrobial Effect against	References
*Latilactobacillus sakei*	Isolated from meat sausages	Garlic powder and wine	*L. monocytogenes*	[62]
Mix of starters	Commercial starter cultures	Mustard	*L. monocytogenes*	[63]
			*E. coli*	
*Latilactobaciullus sakei*	Isolated from meat sausages	Garlic powder and wine	*Salmonella spp.*	[62]
*Latilactobaciullus sakei*	Isolated from meat sausages	Essential oils	*Salmonella spp*	[64]
			*E. coli*	
			*L. monocytogenes*	
Mix of *S. xylosus* and *Lactiplantibacillus plantarum*	Isolated from meat sausages	Vaccum packagaing	*Enterobacteriaceae*	[65]

The nomenclature of the genus Lactobacillus was presented according to the new taxonomic classification [83].

**Table 3:** Antimicrobial effect of selected starter cultures (added as ingredients during cheese manufacture) against main foodborne pathogens.

Cheese	Starter(s) Culture(s) Used	Origin of Starter Cultures	Characterisation of the Inhibition Mechanism	Reference
(a) Starter cultures selected against *L. monocytogenes*				
Anari cheese	*Enterococcus faecium*	Donkey milk	Not determined	[149]
Cottage cheese	*Lactococcus lactis*	Italian fermented food	Nisin producer. PCR detection of bacteriocin genes	[134]
Portuguese Pico cheese	*Lactococcus lactis* *Enterococcus faecium*	Isolated from cheese	PCR detection of bacteriocin genes	[150]
Fresh Minas cheese	*Lactiplantibacillus plantarum* 59	Isolated from fruits	Not determined	[151]
Munster cheese	*Enterococcus faecium WHE 81*	Isolated from cheese	Enterocin producer. Determination by sensitivity to proteolytic and other enzymes	[124]
Fresh cheese	*Lactococcus lactis*		Nisin producer. Bacteriocin gene determination	[152]
Cheese model	*Lactiplantibacillus plantarum*	Isolated from cheese	Plantaricin producer. Purification by HPLC	[153]
Gongonzola Cheese (Italy)	*Lactiplantibacillus plantarum* *Latilactobacillus sakei* *Lactococcus lactis*	Microbial collection (Institute of 108 Sciences of Food Production of the National Research Council of Italy	Nisin and enterocin P producers. Characterisation by bacteriocin gene identification	[123]
Fresh minas cheese	*Enterococcus mundtii* *Enterococcus faecium CRL 35*	Isolated from cheese	Enterocin identification by HPLC and sensitivity to proteolytic and other enzymes	[154]
Ripened cheese	*Pediococcus acidilactici* 347 *Lactococcus* *lactis* ESI 515 *Lactococcus* *lactis* CL1 *Lactococcus* *lactis* CL2	Isolated from dairy products	Nisin and pediocin producers	[121]
Sicilian cheese	*Lactococcuslactis*, 623 *Lacticaseibacillus rhamnosus* 971 *Enterococcus faecium*	Isolated from dairy environment	Not determined	[155]
Golka cheese	*Lactococcus garvieae* Lab428 *Lactococcus mesenteroides* Lab25 *Lactiplantibacillus* *plantarum* Lab572	Isolated from Golka cheese	Characterisation by bacteriocin gene identification	[125]
(b) Starter cultures selected against *Staphylococcus aureus*				
Probiotic white cheese	Commercial lyophilised starter culture *Lacticaseibacillus* *rhamnosus* *Lacticaseibacillus casei* Shirota	Commercial starter cultures	Not determined	[126]
Commercial cheese	*Lactococcus lactis* L005 *Lacticaseibacillus* *rhamnosus* BGP2 *Brevibacterium linens* 004-0001 *Microbacterium lacticum*	Isolated from raw milk	Not determined	[135]
Ripened cheese	*Pediococcus acidilactici* 347 *Lactococcus* *lactis* ESI 515 *Lactococcus* *lactis* CL1 *Lactococcus* *lactis* CL2	Isolated from dairy products	Nisin producer Pediocin producer	[121]
Raw milk Montasio cheese	*Lactiplantibacillus plantarum*	Commercial starter mix	Not determined	[145]
Algerian´s goat cheese	*Lactococcus lactis* ssp. *lactis* KJ660075 strain	Isolated from raw goat milk	Detection of bacteriocin by sensitivity to proteolytic and other enzymes	[156]
(c) Starter cultures selected against *Escherichia coli*				
Jben (Moroccan fresh cheese)	*Lactococcus lactis* subsp. *lactis* UL730	Not available	Nisin producer	[138]
Commercial cheese	*Lactococcus lactis* L005 *Lacticaseibacillus* *rhamnosus* BGP2 *Brevibacterium linens* 004-0001 *Microbacterium lacticum*	Isolated from raw milk	Not determined	[135]
Ripened cheese	*Pediococcus acidilactici* 347 *Lactococcus* *lactis* ESI 515 *Lactococcus* *lactis* CL1 *Lactococcus* *lactis* CL2	Isolated from dairy products	Nisin producer Pediocin producer	[121]
(d) Starter cultures selected against *Salmonella spp.*				
Goat cheese	Authochthonous *Lactobacillus* spp.	Raw goat milk	Not determined	[146]
Raw milk Montasio cheese	*Lactiplantibacillus plantarum*	Commercial starter cultures	Not determined	[145]
White Brined Cheese	*Streptococcus thermophilus* *Lactobacillus delbrueckii* subsp. *bulgaricus* *Lacticaseibacillus paracasei* K5	Isolated from Greek Feta cheese	Not determined	[147]

The nomenclature of the genus Lactobacillus was presented according to the new taxonomic classification [83].

## Data Availability

Not applicable.
